# Strengthening the efficacy of official food control improves *Listeria monocytogenes* prevention in fish-processing plants

**DOI:** 10.1038/s41598-018-31410-9

**Published:** 2018-08-30

**Authors:** Mariella Aalto-Araneda, Hannu Korkeala, Janne Lundén

**Affiliations:** Department of Food Hygiene and Environmental Health, Faculty of Veterinary Medicine, University of Helsinki, P.O. Box 66 (Agnes Sjöbergin katu 2), 00014 University of Helsinki, Helsinki, Finland

## Abstract

Vacuum-packaged cold-salted and cold-smoked fish products are considered typical vehicles for *Listeria monocytogenes*, the causative agent of the food-borne disease listeriosis, which is increasingly prevalent in the European Union. Efficacy of both the fish processing plant self-checking system and official food control conducted by authorities are crucial for *L*. *monocytogenes* prevention in the processing of these risky products. However, the impact of official control on *L*. *monocytogenes* prevention in the processing of fish products has not been extensively studied. We investigated the occurrence, control measures, and correction of non-compliances predisposing to *L*. *monocytogenes* in Finnish fish processing plants. The following features were associated with *L*. *monocytogenes* occurrence: (a) frequency of non-compliances concerning processing machinery, (b) recurrence of non-compliances, and (c) frequency of non-compliances for which official control measures were requested by inspecting authorities. Official control of fish processing plants had focused on risky areas, but non-compliances were common and their correction exhibited deficiencies. We conclude that *L*. *monocytogenes* prevention in fish processing can be enhanced by strengthening official food control measures and processing plant compliance. In particular, timely correction of all food safety violations must be improved.

## Introduction

Increased incidence of the severe food-borne disease listeriosis has been reported in Finland and elsewhere in Europe in the 2010s^1,2^. Control measures have thus been insufficient, and foodstuffs contaminated with *Listeria monocytogenes* are found on the market. Seafood, in particular, are considered typical vehicles, and ready-to-eat vacuum-packaged gravad (cold-salted) and cold-smoked fish products have been implicated in listeriosis outbreaks^3–5^. In the most recent investigations by Finnish public food authorities, the prevalence of *L*. *monocytogenes* in this type of fish products ranged from 12 to 32%^6,7^, and the bacterium was occasionally found from facilities and food contact surfaces of fish processing plants^8^. Violations in hygiene practices, maintenance, and cleanliness have been reported as drivers of *L*. *monocytogenes* contamination^9,10^. Stringent interventions targeting such violations have proven successful at reducing *L*. *monocytogenes* occurrence in fish processing environments^11,12^. Thereby, an efficient self-checking system alongside adequate official control measures are crucial for *L*. *monocytogenes* prevention in the production of these risky products.

In Finland, the official food control system is based on European Union food safety legislation complemented by a national act of legislation and decrees notified to the EU. The entire food safety field is overseen by the Finnish Food Safety Authority (Evira) working under the Finnish Ministry of Agriculture and Forestry. Authorities at regional administrative offices supervise and audit the municipal food control units, where official food safety inspectors are employed. Fish-processing plant inspections are carried out by the municipal authorities. If non-compliances of food safety legislation are observed, inspectors can give advice, demand corrections, or use enforcement measures to remove the observed violations^13^. The execution of official control must be regular, risk-based, and sufficiently frequent^14^. Producing vacuum-packaged gravad or cold-smoked fish is considered a highly risky operation requiring 5 to 11 annual inspections^15^.

Implementation of food safety practices has been shown to necessitate improvement among some fish industry operators^16^. Food business staff and management may also underestimate food safety risks^17,18^. Reportedly, Finnish fish processing plants perceive the food safety risks related to their operations smaller than other food processing plants^19^, although the processing of vacuum-packaged ready-to-eat fish products evidently contains a risk of *L*. *monocytogenes*. In order to elucidate the current state of listeria-related food safety violations and efficacy of official control, we conducted an investigation of ready-to-eat fish processing plants in Finland.

Our aim was to study the efficacy of official control pertaining to occurrence, control measures, and correction of non-compliances predisposing to *L*. *monocytogenes* in Finnish food business operators producing gravad and cold-smoked vacuum-packaged fish. Prior to our investigation, no detailed overview was available on the efficacy of official food control in ready-to-eat fish production. With statistical modelling, we identified features of official control and fish-processing plant compliance that associated with *L*. *monocytogenes* occurrence, such as deficiencies in processing machinery and correction of violations. Our results highlight ways of improving official food control that support *L*. *monocytogenes* prevention in the fish industry.

## Material and Methods

### Fish processing plants and official inspections

From all over Finland, 21 fish-processing plants (FPPs) producing vacuum-packaged gravad and/or cold-smoked fish participated in this investigation (Table 1). According to the listing of the Finnish Food Safety Authority Evira, our sample included a third of the Finnish FPPs producing this type of products during the study period. Upon request to provide official inspection records of each FPP from the previous three years, the inspectors delivered documentation covering on average 2.6 years of food control inspection reports and official microbiological sampling certificates, encompassing years 2011–2014. Data arising from these inspection records were used to retrospectively determine the occurrence of *L*. *monocytogenes* in the facilities and products of each FPP from January 2011 to December 2013. Inspected items, including non-compliances and official control measures, were also extracted from inspection reports (see section “Data analysis of official inspection reports”).

**Table 1:** *Listeria monocytogenes*, inspections, and non-compliance occurrence in fish processing plants.

Fish processing plant	Total amount of *Lm*^a^-positive samples in 2011–2013 (of which *Lm* -positive RTE^b^ products)	Production (“1” over 100 tons; “2” 100 tons or under)	Planned amount of inspections in 2014 (% of FFSA^c^ recommendation)	% of planned inspections realized in 2014	% of inspections occurred during processing in 2014	% of NCs^d^ from inspected items
A	14 (2)	1	10 (200)	80	50	68
B	8 (3)	1	8 (73)	50	75	59
C	8 (2)	1	8 (73)	100	100	69
D	5 (0)	1	4 (80)	75	100	71
E	3 (0)	1	11 (100)	91	100	61
F	2 (1)	1	7 (64)	100	43	38
G	2 (1)	2	5 (100)	60	100	56
H	2 (1)	1	8 (73)	88	100	26
I	2 (0)	2	5 (100)	60	67	29
J	0 (0)	1	8 (160)	63	100	50
K	0 (0)	1	5 (45)	80	100	67
L	0 (0)	2	4 (80)	125	60	15
M	0 (0)	1	2 (40)	50	0	23
N	0 (0)	2	5 (100)	60	0	41
O	0 (0)	1	5 (45)	80	25	39
P	0 (0)	2	3 (60)	67	50	63
Q	0 (0)	2	5 (100)	80	50	39
R	0 (0)	2	5 (100)	60	100	45
S	0 (0)	2	2 (40)	100	0	64
T	0 (0)	1	10 (200)	80	50	50
U	0 (0)	2	4 (80)	100	75	33

^a^*Lm* = *Listeria monocytogenes*; ^b^RTE = ready-to-eat; ^c^FFSA = Finnish Food Safety Authority*;* ^d^NCs = non-compliances.

In addition, we estimated the efficacy of municipal official food control through fulfilment of Finnish Food Safety Authority Evira requirements on number of annual inspections^15^, execution of planned inspections, and proportion of annual inspections carried out during processing. The amount of inspection reports obtained per FPP varied from 2 to 27 (median 9), and their quality varied between inspectors from detailed to cursory. In five FPPs, inspection reports were noticed missing because of discontinued content or reported missing by the inspectors due to either FPP moving from under the jurisdiction of one municipality to another, municipal control unit changing their data storage system, or irregular documentation due to lack of personnel. Thereby, calculation of the annual number of inspections for all FPPs was not possible from the inspection reports obtained. The planned and executed inspections, and inspections carried out during processing, were therefore inquired from the inspectors via an online questionnaire for the year 2014, for which these data could be reliably obtained during conduction of this study in 2015.

### Data analysis of official inspection reports

The official inspection reports were read by one researcher (MA-A), and inspected items (n = 2803) were extracted into an Excel-file. Each item was assigned the following initial variables: (a) FPP, (b) date, (c) sector of FPP self-checking system (Table 2), and (d) non-compliance (no–yes). For the items classified as non-compliances, the following additional variables were allocated: (e) whether the non-compliance related to plans, documentation, or facilities and operations, (f) whether the non-compliance concerned processing equipment (no–yes), (g) type of control measure (none–advice–demand for correction–enforcement measure), (h) written time limit given for correction (none–days), (i) re-inspection of non-compliance (no–yes), (j) re-inspection within given time limit (no–yes), (k) correction of non-compliance (no–partly–yes), (l) correction of non-compliance within given time limit (no–partly–yes), and (m) recurrence of non-compliance (no–yes). In addition, it was noted for each FPP, whether the inspection reports mentioned problems in collaboration, such as FPP withholding information or criticizing the inspector.

**Table 2:** The occurrence of inspected items and non-compliances (NCs) by sectors of fish processing plant (FPP) self-checking system.

Sector of FPP self-checking system	Inspected in % of FPPs (n = 21)	NCs in % of FPPs where sector inspected	No. of inspected items (% of total n = 2802)	% of NCs out of inspected items	“Indirect” *Lm*-risk^e^ in % of NCs	“Direct” *Lm*-risk^f^ in % of NCs
Processing hygiene	100	81	326 (12)	53	45	35
Sampling and samples^a^	100	90	303 (11)	42	25	32
Sanitation	95	95	361 (13)	55	53	28
Suitability of premises and equipment	100	81	203 (7.2)	43	50	26
Working hygiene	90	84	142 (5.1)	56	48	24
Waste water management^b^	33	100	23 (0.8)	83	47	21
Traceability	67	72	84 (3.0)	55	39	15
Raw material reception	71	61	48 (1.7)	52	36	12
Handling of waste and by-products	86	60	58 (2.1)	45	58	12
Temperature control	95	85	214 (7.6)	48	56	7.8
Self-checking system plan^c^	95	75	124 (4.4)	56	48	7.2
Maintenance	90	96	259 (9.2)	65	50	5.3
Pest control	71	73	37 (1.3)	51	53	5.3
Approval of establishments	81	53	70 (2.5)	39	0.0	3.7
Package labelling	90	100	86 (3.1)	72	16	3.2
Orderliness	95	85	201 (7.2)	62	71	1.6
Management of foreign substances	76	75	42 (1.5)	48	0.0	0.0
Sending and transport of products	62	69	42 (1.5)	41	0.0	0.0
Management of special circumstances^d^	62	47	28 (1.0)	25	0.0	0.0
Management of product composition	52	63	32 (1.1)	38	0.0	0.0
Management of contact materials	52	56	24 (0.9)	50	0.0	0.0
Verification of hygiene know-how	52	46	24 (0.9)	42	60	0.0
Follow-up of health condition	52	46	19 (0.7)	32	0.0	0.0
Hygiene education	52	46	21 (0.7)	29	50	0.0
Parasite inspections	24	79	14 (0.5)	50	0.0	0.0
Management of allergens	19	53	12 (0.4)	17	0.0	0.0
Fish freezing requirements	14	71	5 (0.2)	40	0.0	0.0

^a^Sampling plans and samples for microbiological quality and process hygiene, e.g. pathogens, indicators, histamine and PAH compounds.

^b^Waste water management: melting waters, water used in processing, removal of water used in cleansing, sewage.

^c^Self-checking system plan: adequacy of plan at general level, inclusion of required sections.

^d^Management of special circumstances: preparedness and handling of product withdrawals, customer complaints etc.

^e^Non-compliance indirectly predisposes to *Listeria monocytogenes* spread, growth, or contamination, see Supplementary Table S1.

^f^Non-compliance directly predisposes to *L*. *monocytogenes* spread, growth, or contamination, see Supp. Table S1.

### Classification of non-compliances

The inspected items were classified into 27 sections describing the scope of FPP procedures (i.e. sectors of FPP self-checking system, Table 2). The classification was based on the regulation for food hygiene of approved food establishments^20^ and the grouping (table of contents) used in the guidelines of the Finnish “Oiva” system for official food control^21^. The link of each non-compliance with *L*. *monocytogenes* spread, growth, or contamination was assessed at a 3-level scale (“no risk” – “indirect” – “direct”, Supp. Table S1) based on known contamination patterns and risk factors reported in literature^5,9,11,12,16,22–31^.

### Statistical analyses

Univariate analyses concerning non-compliances and comparison of FPPs, where *L*. *monocytogenes* occurred (“listeria-positive”) or did not occur (“listeria-negative”), were performed in SPSS version 25. The usage between parametric and non-parametric tests was based on Shapiro-Wilk’s test of normality.

The quantitative data describing percentages of non-compliances and control measures for each FPP were used in a generalized linear model (GLM) to explain the number of times *L*. *monocytogenes* occurred in facilities and products during 2011–2013. GLM was carried out in R version 3.4.0^32^ using ‘*glm’* (family = ‘poisson’ and ‘quasipoisson’, link = ‘log’) following the data exploration, analysis, and variable selection protocol described by Zuur *et al*. (2009 and 2010)^33,34^. Collinearity was checked using Pearson and Spearman correlation coefficients and Variance Inflation Factor (VIF): only variables with VIF < 3 were included in the model fitting^34^. The model was initially fitted with eight covariates (Supp. Table S2), including (a) FPP output (tons), (b) proportion (%) of non-compliances out of items inspected, (c) proportion (%) of non-compliances predisposing indirectly or directly to *L*. *monocytogenes*, (d) proportion (%) of non-compliances given time limit for correction, (e) proportion (%) of non-compliances not corrected, (f) proportion (%) of non-compliances recurred, (g) proportion (%) of non-compliances related to processing equipment, and (h) proportion (%) of non-compliances given an official control measure by inspector. Backward selection based on likelihood ratio tests (‘*drop1’*) was used to remove variables while the residual deviance remained insignificant on the 0.05-level^33^. Overdispersion (dispersion 1.5, z = 2.1, p = 0.02) was observed by dispersion test in ‘*AER*’ package^35^ and therefore quasi-Poisson regression was applied for the final model^33^. No observations exceeded Cook’s distance 1 and hence were not deemed influential^36^. Explained deviance (or pseudo-R²^37^) was calculated for the final model, which included intercept and three main effects.

## Results

### Inspection frequency in *L. monocytogenes* positive and negative FPPs

In 2011–2013, *L*. *monocytogenes* had occurred in the products or facilities of 9/21 FPPs (A – I, Table 1), which were classified “listeria-positive”. These listeria-positive FPPs could be divided into two categories: (1) FPPs A, B and C where *L*. *monocytogenes* was recurrently found, and (2) FPPs D–I, where contamination was occasional. FPPs A–C suffered from a continuous listeria problem, where *L*. *monocytogenes* was repeatedly found in products and on food contact surfaces from several rooms and processing machines. This indicates that the contamination was widespread throughout the processing environment and could have been persistent. In FPPs D–I, *L*. *monocytogenes* contamination rarely occurred in products or food contact surfaces and was evidently sporadic. *L*. *monocytogenes* did not occur in FPPs J–U (n = 12, Table 1), which were classified “listeria-negative”.

All inspections planned for 2014 had been executed in only 5/21 FPPs. The planned amount of inspections followed the national guidelines^15^ for the majority of FPPs (Table 1), but in 3/21 it had been increased and in 4/21 decreased more than the 50% recommended. Out of the listeria-positive FPPs, the inspection amount had been decreased in 5/9 and increased in 1/9 (which was the listeria-problematic FPP A); respective numbers for the listeria-negative FPPs were 7/12 and 2/12. All inspections had taken place during production in 8/21 FPPs in 2014, whereas in 3/21 FPPs, official control had not inspected ongoing production at all (Table 1). Inspection during production appeared a more common practice in listeria-positive than listeria-negative FPPs, where on average 82% and 51% of inspections, respectively, took place during production (Mann-Whitney U test, p = 0.08). Based on the amounts of inspected items, inspection efforts had evidently focused on certain sectors of the self-checking system, such as processing hygiene and sanitation, while others, such as fish freezing requirements or allergen management, had not been inspected at all in many FPPs (Table 2).

### Non-compliances predisposing to *L*. *monocytogenes* at different sectors of the self-checking system

Out of the recorded non-compliances (n = 1456) from all studied FPPs, the majority (83%) concerned operations and facilities, whereas 12% concerned plans and 5% documentation. When classified according to sectors of the FPP self-checking system (Table 2), the majority of non-compliances (67%) concerned 7 out of 27 sectors: either sanitation, processing hygiene, maintenance, food hygiene sampling and samples, orderliness, temperature control, or suitability of premises and equipment. Such non-compliances occurred in most of the FPPs (81–90%), as did those related to package labelling.

Notably, non-compliances occurring on 18 out of the 27 sectors of the FPP self-checking system were estimated having potential to predispose “indirectly” or “directly” to *L*. *monocytogenes* spread, growth or contamination (Table 2 and Supp. Table S1). Highest proportions (21–35%) of “direct listeria risk” were observed among non-compliances related to processing hygiene, food hygiene sampling and samples, sanitation, suitability of premises and equipment, working hygiene, and waste water management (Table 2). While frequent among most FPPs, non-compliances potentially posing either an “indirect” or “direct” listeria risk appeared somewhat more common among listeria-positive than listeria-negative FPPs (61% vs. 51%; Student’s t-test, p = 0.09, Table 3).

**Table 3:** Summary of non-compliances (NCs), their official control measures and correction in *Listeria monocytogenes* (*Lm*) positive (n = 9) and negative (n = 12) fish-processing plants (FPPs).

Covariate	Average in *Lm-*positive FPPs (variation among them)	Average in *Lm-*negative FPPs (variation among them)	Student’s t-test (or Mann-Whitney U test*) p-value^b^
% of NCs from inspected items	53 (26–71)	44 (15–67)	0.2
% of NCs posed “indirect” or “direct” *Lm*-risk^a^	61 (35–80)	51 (33–69)	0.09
% of NCs concerned machinery	11 (0–19)	7.8 (0–21)	0.4
% of NCs given time limit	31 (7–80)	20 (0–81)	0.2*
Control measures followed % of NCs	90 (83–96)	94 (84–100)	0.1
% of NCs re-inspected	61 (50–74)	52 (14–83)	0.3
% of NCs not corrected	31 (4–86)	26 (0–100)	0.5
% of NCs recurred	30 (17–39)	23 (0–55)	0.3

^a^NC indirectly or directly predisposes to *Lm* spread, growth, or contamination, see Supplementary Table S1.

^b^Statistical difference between average (or median*) of *Lm*-positive and negative FPPs.

### Official control measures

Non-compliances were typically (78%) followed by a demand for correction and less often (11%) by advice without demands. The proportion of non-compliances followed by a demand for correction varied considerably (43–100%) among FPPs. No request for official control measures was given by the inspector for 9% of the non-compliances, while an additional 1.1% did not require intervention from the inspector as the FPP itself reacted. Enforcement measures had been used once in two FPPs during the investigated period. Re-inspection was mentioned in the official inspection reports for 65% of the non-compliances, and out of these, on average 51% were completely and 18% partially corrected during the study period. A third (33%) of the non-compliances recurred: some up to 16 times but generally two to three times.

Inspectors gave demands for correction more often for non-compliances posing a listeria risk (either “indirect” or “direct”) than for other non-compliances (80% vs. 74%; Fisher’s exact test, p = 0.007), but no significant difference was observed in their correction (72% vs. 73% respectively corrected; Fisher’s exact test, p = 0.8). Furthermore, the higher the deemed listeria-risk was on the scale “no risk–indirect–direct”, the significantly shorter were the time limits given for correction (time limit medians “no risk” 30 d, “indirect” 13 d, “direct” 0 d; Kruskall-Wallis, p < 0.001). However, no significant differences were observed in the correction of non-compliances with regard to presence or absence of time limits (73% vs. 71% respectively corrected; Fisher’s exact test, p = 0.5).

In 3/9 of the listeria-positive FPPs, all reported *L*. *monocytogenes* findings had led to corrective actions initiated by the FPP and required no further intervention from official control, whereas at another 3/9 FPPs, corrective actions for *L*. *monocytogenes* were in all cases initiated by a demand for correction from the inspector. In FPPs A, B and C where *L*. *monocytogenes* most frequently occurred, 88–100% of *L*. *monocytogenes* findings required demands from inspectors to initiate the control measures.

### Factors associated with *L*. *monocytogenes* occurrence and widespread contamination

Summary of variables describing non-compliances and their control measures in listeria-positive and negative fish-processing plants is included in Table 3, but no statistical differences were observed in these univariate analyses. However, generalized linear modelling (quasi-Poisson regression) was performed to identify covariates (Supp. Table S2) explaining the number of times *L*. *monocytogenes* occurred in the FPPs during 2011–2013 (varying from 1 to 14 times, Table 1). According to the model, the expected occurrence of *L*. *monocytogenes* in an FPP grew higher when larger proportions of their non-compliances (1) recurred, (2) concerned processing machinery, and (3) did not result in inspectors requesting official control measures (Table 4). These covariates were highly significant and based on all non-compliances at a particular FPP – not only those related to *L*. *monocytogenes*.

**Table 4:** Generalized linear model (quasi-Poisson regression, pseudo-R^2^ = 65%) for covariates associated with increase in *Listeria monocytogenes* occurrence in fish processing plants (n = 21).

Model attribute or parameter	Deviance	Df	F test	p value	Estimate	Std. error
Null deviance	97.7	20				
Residual deviance	34.2	17	63.5	<0.001		
Intercept					6.63	3.71
% of non-compliances related to processing machinery		1	26.4	<0.001	0.21	0.05
% of non-compliances given an official control measure by inspector		1	6.48	0.02	−0.13	0.05
% of non-compliances recurred		1	15.1	0.001	0.11	0.03

Df = degrees of freedom; F = F test statistic for residual deviance.

Dirtiness, poor condition of surfaces, and *L*. *monocytogenes* findings in processing machines were repeatedly pointed out in inspection reports of FPPs A–C with widespread contamination despite stated efforts of intensified sanitation. However, enforcement measures were not applied to tackle the recurring hygienic non-compliances. According to the inspection report findings, particularly problematic areas were head removal, filleting, and trimming lines. For instance in FPP A, the contaminated part of a fish bone removal machine was identified using extensive sampling. However, the inspector pointed out non-compliances of this machine on three separate occasions over the course of a year before the FPP replaced the contaminated part, and subsequently, contamination was no longer detected in the machine. Thereafter, contamination appeared in skinning and slicing machines of this FPP.

Problems in collaboration between FPP and inspector were noted only in inspection reports of FPPs A–C with widespread contamination. These issues displayed as withholding information on *L*. *monocytogenes* positive samples from the authorities, disputing the actions of the inspector, denying or downplaying non-compliances, and taking the concerns of the inspector as accusations resulting in the FPP taking a defensive position. Similar issues were not documented from FPPs D–U.

## Discussion

The planned number of inspections had been reduced from that recommended by the Finnish Food Safety Authority in many listeria-positive FPPs. In 2011–2014, the annual amount of inspections was based on national guidelines, which the food control unit could decrease or increase by 50% according to their own risk assessment^15^. In relation to our results, the annual amount of official risk-based inspections should reflect the inspection history of the FPP including the extent of non-compliances occurring in processing machinery and on sectors of the self-checking system most associated with listeria risk. Reducing inspections in FPPs with sustained *L*. *monocytogenes* occurrence seems illogical but may imply inadequate or poorly organized resources. Indeed, all planned annual inspections were completed in only few of the studied FPPs. Control objects per personnel work year vary between municipal food control units, and several food control officials have reported insufficient personnel resources^38^. Moreover, inspections had always taken place during processing in less than half of the studied FPPs, while according to our findings, non-compliances with listeria risk often involved concrete operations. Effort should be made to inspect each FPP during processing^14^, which must be applied particularly stringently to FPPs with known history of *L*. *monocytogenes*. Risk-based official control should also lean on former inspection findings^14,39^, which may not have been possible in FPPs where we found parts of the control history missing or inspection reports cursory. Using inspection report templates has been shown to improve their quality and efficacy^40^. Since 2015, after transition to the official food control system “Oiva”, all inspections in Finland are documented with a systematic template into an online database, where also summaries of inspection findings are publicly available^21^. Our data and results can be used as a reference when evaluating the efficacy of the reformed national control system in the future.

Finnish FPPs have stated they are able to correct the food safety issues pointed out by inspectors^41^ and regard their food safety risks smaller than other food processors^19^. Conversely, our results show that non-compliances were common among the studied FPPs, and majority of them could predispose to *L*. *monocytogenes*. Fish industry operators worldwide face similar challenges, and several factors affecting *L*. *monocytogenes* contamination in fish processing have been identified^5,9,11,12,16,27,42^. Interestingly, in a study performed in the smoked fish industry in Spain, majority of non-compliances noted by official control were evaluated not to affect the safety of the product^43^. Our results support an opposite view: most sectors of the FPP self-checking system were estimated to influence *L*. *monocytogenes* spread, growth or contamination, if non-compliances occur. Listeria risk was prevalent particularly among non-compliances related to processing and working hygiene, food hygiene sampling and samples, sanitation, suitability of premises, orderliness, and maintenance. Non-compliances also most frequently – and often simultaneously – occurred in the aforementioned sectors of the self-checking system. Lundén (2013) also reported multiple concurrent non-compliances in fish processing plants, several of which could predispose to outbreaks^44^. In the current study, prevalence of non-compliances predisposing to *L*. *monocytogenes* was somewhat higher in listeria-positive than negative FPPs. Manifestation of such non-compliances in listeria-negative FPPs, however, indicates that not only the presence of non-compliances but also practices of dealing with them can explain the differences in *L*. *monocytogenes* occurrence.

The sectors of the self-checking system where majority of non-compliances predisposing to *L*. *monocytogenes* concentrated, were also the sectors inspected more often than others. Thus, inspectors seem to have focused on risky areas, where violations were common. They had evidently also assessed the risks of non-compliances: correction was more often demanded and shorter time-limits given to non-compliances we estimated predisposing to *L*. *monocytogenes* to a greater extent. However, correction rates were similar despite estimated listeria risk, and in many FPPs, overall non-compliance recurrence was relatively high and correction rate low. Official control measures consisted mainly of demands for correction, which were generally not strengthened by enforcement measures. The infrequent use of enforcement measures by food control authorities has been discussed by several studies^41,44–46^. Although the enforcement processes can sometimes be lengthy, compliance has been reported for the majority of violations indicative of their utility for prolonged non-compliances^45^. Recurrence may also reflect more the difficulty FPPs experience with non-compliances than imply inefficacy of time limits or demands, as time limits have previously been associated with successful correction and smaller occurrence of violations^40,46^. Long enough time periods may be important for correction^40^, and thereby, time limits should consider both the FPP resources and the severity of the non-compliance. Ideally, re-inspection should be carried out within the time limit to ensure its efficacy. A protocol for using time limits and re-inspections for all observed non-compliances was recently introduced by the Finnish food control system reform “Oiva”, where follow-up inspections and enforcement measures ensue until non-compliances impairing or jeopardizing food safety have been corrected^21^. The potential of the reformed system in strengthening the elimination of violations requires further investigation.

In this study, FPPs had either no occurrence of *L*. *monocytogenes* (FPPs J–U), sporadic findings (FPPs D–I), or a continuous *L*. *monocytogenes* problem (FPPs A–C). Since raw fish occasionally contains *L*. *monocytogenes*, it is likely that contamination sometimes occurs^26,47–50^. However, persistent contamination often develops in the processing environment and machinery, through which widespread contamination of the end products may ensue^22,24,51–53^. Stringent cleaning has proven successful in eliminating contamination^11^, but can only be performed if surfaces remain in good condition. Dirty processing machinery in poor condition undoubtedly played a role in sustaining a continuous, potentially persistent contamination at FPPs A–C. Despite a relatively small sample size for statistical power, we were able to connect *L*. *monocytogenes* occurrence at FPPs to the functioning of their self-checking systems and official control. As we exhibited through generalized linear modelling, the larger the proportion of processing-machine-related non-compliances was, the higher was the *L*. *monocytogenes* occurrence in the FPPs. Strengthening the self-checking system and official inspection efforts for cleanliness, maintenance, and hygienic handling of the processing machines can help reduce the amount of *L*. *monocytogenes* findings particularly in FPPs with potentially persistent contamination.

Our results also show that *L*. *monocytogenes* occurrence correlates positively with recurring non-compliances and inversely with those followed by inspector requesting official control measures. This indicates that efficiency in handling all types of non-compliance can reflect upon *L*. *monocytogenes* control. If correction is not required for all violations, FPPs may develop a careless attitude even towards the severe ones. We observed that FPPs A–C suffering from continuous listeria problems and widespread contamination demonstrated mistrust against official control, which was not observed in FPPs D–I with occasional contamination nor in listeria-negative FPPs J–U. This finding indicates that lack of mutual understanding may have been at the root of some problems, and FPPs with continuous, potentially persistent contamination may need more encouragement and building of trust from the part of the inspecting authorities. The attitudes of food business operators have been shown to affect processing hygiene and relationship with authorities^54–56^. Better understanding of food safety risks and how they reflect upon the business can help appreciate official control^57^, but knowledge alone may not lead to change of behaviour in food handlers, if management, resources and infrastructure do not support it^18^. Since listeria control depends on continuous hygienic practices, it needs to be incorporated into everyday routines, and employees must understand its importance^58^. Professionalism and ability to negotiate from the part of the inspector motivates food business operators to perform the demanded corrections^57^. Thereby, opportunities for participation in advanced food control training must be ensured for all FPP inspectors: training on building rapport, generating motivation, facilitating behavioural change, and achieving compliance through persuasive communication could be useful for dismantling frustrating situations, which listeria problems and recurring non-compliances often are.

Our main findings indicate that the higher the *L*. *monocytogenes* occurrence is, the more important it is to reduce non-compliances related to processing machinery, demand correction of all non-compliances and prevent their recurrence. Both current EU legislation^14,59^ and rules entering into application in December 2019^60^ require verification of efficacy and corrective actions from risk-based official control, and oblige the inspection of hygiene conditions, food processing, machinery, and cleaning and maintenance procedures. Thereby in light of our results, improving *L*. *monocytogenes* prevention by means of official control pertains more to enhanced implementation than amendments to the legislative framework itself. History of non-compliances regarding machinery, hygiene, and sanitation should lead to increased inspection frequency by food control authorities. Timely correction of violations should always be demanded, and efficient methods ensuring lasting removal of non-compliances need to be developed and adopted. These could include establishing a systematic re-inspection protocol such as the “Oiva” system^21^, improving supervision of time limits, enhancing collaborative communication between FPPs and inspectors, and opportune use of enforcement measures.

## Conclusions

Our study was the first in-depth investigation on the efficacy of official control in the production of ready-to-eat fish products. We identified control efforts that need improvement and showed that inefficiency of official control can associate with poor *L*. *monocytogenes* prevention. Non-compliances predisposing to *L*. *monocytogenes* were common in many fundamental operations of FPPs and particularly in processing machinery. Official control had focused inspection efforts and control measures towards risky operations, but non-compliances recurred relatively often despite demands for corrections and given time limits, which indicated insufficiency of current methods and communication. *L*. *monocytogenes* occurrence can be reduced by always requiring official control measures for non-compliances as well as applying more stringent surveillance of their execution, paying particular attention to non-compliances regarding machinery, hygiene, and sanitation. Recommendations based on our results are applicable to fish processing as well as other food industry areas where *L*. *monocytogenes* control is crucial.

## Electronic supplementary material

Supplementary Table S1

Supplementary Table S2

## Data Availability

Data used for generalized linear modelling are included in this published article and its Supplementary Table S2. The inspection records that support the findings of this study can be requested from the municipal food control units but cannot be shared by the researchers without breaching anonymity of the study participants. Other datasets generated and analysed during the current study are also not publicly available due to confidentiality agreements with the participants of this study.
